# Does Electrical Stimulation through Nerve Conduits Improve Peripheral Nerve Regeneration?—A Systematic Review

**DOI:** 10.3390/jpm13030414

**Published:** 2023-02-26

**Authors:** Sophie Hasiba-Pappas, Lars-P. Kamolz, Hanna Luze, Sebastian P. Nischwitz, Judith C. J. Holzer-Geissler, Alexandru Cristian Tuca, Theresa Rienmüller, Mathias Polz, Daniel Ziesel, Raimund Winter

**Affiliations:** 1 Research Unit for Tissue Regeneration, Repair and Reconstruction, Division of Plastic, Aesthetic and Reconstructive Surgery, Department of Surgery, Medical University of Graz, Auenbruggerplatz 5, A-8036 Graz, Austria; 2 COREMED—Cooperative Centre for Regenerative Medicine, Joanneum Research GmbH, Neue Stiftingtalstr. 2, A-8010 Graz, Austria; 3 European Testing Center of Medical Devices, Institute of Health Care Engineering, Graz University of Technology, Stremayrgasse 16/II, A-8010 Graz, Austria

**Keywords:** nerve regeneration, electrical stimulation, nerve conduit, peripheral nerve injury, sciatic nerve, plastic surgery

## Abstract

Background: Peripheral nerve injuries affect over 2% of trauma patients and can lead to severe functional impairment and permanent disability. Autologous nerve transplantation is still the gold standard in the reconstruction of nerve defects. For small defects, conduits can be considered for bridging. Lately, the combined use of conduits and electrical stimulation has gained attention in the treatment of peripheral nerve injury. This review aimed to present the currently available data on this topic. Methods: PubMed, Embase, Medline and the Cochrane Library were searched for studies on electrical stimulation through nerve conduits for nerve defects in in vivo studies. Results: Fifteen studies fit the inclusion criteria. All of them reported on the application of nerve conduits combined with stimulation for sciatic nerve gaps in rats. Functional, electrophysiological and histological evaluations showed improved nerve regeneration after electrical stimulation. High variation was observed in the treatment protocols. Conclusion: Electrically stimulated conduits could improve peripheral nerve regeneration in rat models. The combined application of nerve guidance conduits and electrical stimulation shows promising results and should be further evaluated under standardized conditions.

## 1. Introduction

Peripheral nerve injuries show a high prevalence and occur in 2–3% of all trauma patients [1,2]. They pose a great challenge for healthcare institutions and, most importantly, put a severe burden on affected patients, many of whom suffer from life-long disabilities due to permanent nerve damage [3].

After nerves are severed due to traumatic injuries, the fibers of the distal nerve stump degenerate through Wallerian degeneration. Neuronal contact is crucial in the recovery process and a lack of it eventually leads to atrophy of the supporting Schwann cells, which can then no longer provide axonal regeneration [4]. Even though some nerve injuries can heal on their own, surgical treatment is necessary in most cases [5]. Insufficient or failed regeneration hinders communication between the peripheral nervous system and the central nervous system (between the brain and spinal cord). Subsequent neuroma formation of the proximal nerve stump and atrophy of the innervated muscles cause further impairment and can lead to neuropathic pain, putting a harrowing strain on the daily lives of affected people [5]. With that in mind, optimizing therapeutic methods to achieve the best possible outcome should be the goal.

Surgical repair of the severed nerves via coaptation is not always a suitable option [6]. Segmental nerve defects could result from nerve crushing, avulsion or shortening of poorly perfused portions of the nerves. Such defects must be bridged. Autologous nerve grafting is currently considered the gold standard to bridge defects of peripheral nerves; however, it is associated with donor site morbidity, issues with size mismatch and neuroma formation [7,8]. Nerve guidance conduits (NGCs) present an appealing alternative to the direct repair of autologous nerve grafts and provide guidance in axonal repair between the proximal and distal nerve stump [9]. The efficacy of synthetic NGCs was reported in the literature [10]; however, the lack of degradability of these materials was disputed as a potential drawback and promoted interest in biodegradable polymers [9,11,12,13,14]. Due to extensive research over the past few decades, a wide range of highly adapted nerve conduits with different materials and properties is available today [15,16,17,18,19].

The use of electroceuticals in peripheral nerve injury (PNI) treatment has been explored for years [20]. While the application of percutaneous electrical stimulation (ES) is quite established in clinical settings [21], direct ES treatment on injured nerves is a far less explored approach, and data on this subject, especially concerning in vivo experiments, is limited. However, various beneficial effects were observed in animal studies, especially with low-frequency ES [7,22,23].

We present a systematic review that (1) displays the currently available studies on ES through nerve conduits for peripheral nerve injuries in animal models, (2) provides information on study protocols and outcomes of in vivo application and (3) interprets the findings and discusses the potential outlook for the future.

## 2. Materials and Methods

### 2.1. Literature Search

The databases Medline, Embase, Cochrane (all via Ovid) and PubMed were searched for studies on the combined application of nerve conduits and electrical stimulation. A systematic search according to PRISMA guidelines was performed from the 1st until the 15th of November 2022. The systematic review protocol for this study was registered on “Inplasy” (registration number INPLASY202320057).

The following terms were applied for the literature search: “nerve conduit” (1), “nerve guidance conduit” (2), “electrical stimulation” (3), “electrically conducting” (4) and “electrically conductive” (5). The details of the search strategy are portrayed in Table 1.

### 2.2. Inclusion and Exclusion Criteria

The search focused on studies that investigated the effects of electrical stimulation combined with nerve conduits in peripheral nerve defects. Publications that only applied either ES or NGCs separately were excluded. Furthermore, trials that reported the use of conductive material (scaffolds or conduits) without the application of therapeutic electrical stimulation did not qualify. To focus on potentially clinically relevant outcomes, trials that were only performed in vitro with no experimental section in vivo were not included. Research concerning the central nervous system, such as spinal cord injuries, was not explored in this review.

### 2.3. Data Extraction

**Figure 1:**
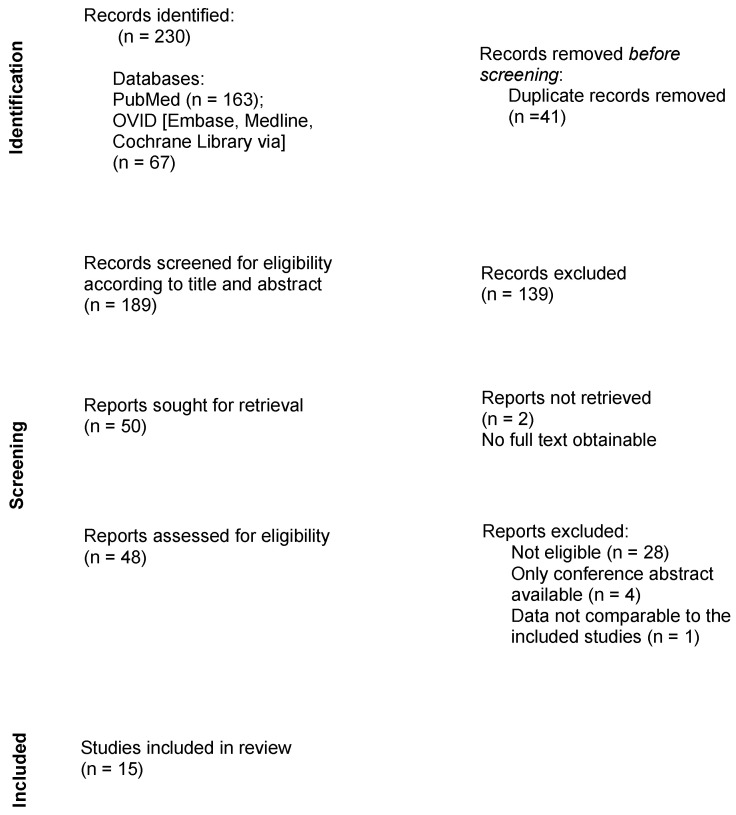
Flow diagram describing the review process.

Following the assembly of the findings of all searched databases, duplicates were manually removed. Titles and abstracts were screened for eligibility, followed by a full review of the remaining publications. The study selection process is presented in the flowchart in Figure 1.

## 3. Results

In total, 230 results were obtained during the research process. No additional records were identified through other sources. After removing duplicates, 189 studies remained. Fifteen publications met the criteria and were therefore included in this systematic review.

All included studies investigated the effect of nerve guidance conduits combined with electrical stimulation on the sciatic nerves of rats. For this purpose, the sciatic nerves were surgically exposed and resected, thereby creating a nerve defect. The exact location of this defect was only reported in a few studies and showed high variability (e.g., described as “mid-thigh”, “lateral thigh” or even just “lower limb”). The length of the nerve gap varied between 2 and 20 mm in the studies. The “study population” consisted of newborn or adult rats, depending on the trial. Most investigators used male rats for the experimental section, while few studies were conducted on female rats.

The authors stated that they had performed the experiments according to ethical animal care guidelines.

### 3.1. NGC Types and ES Protocols

Various materials were used to prepare the nerve guidance conduits.

**Figure 2:**
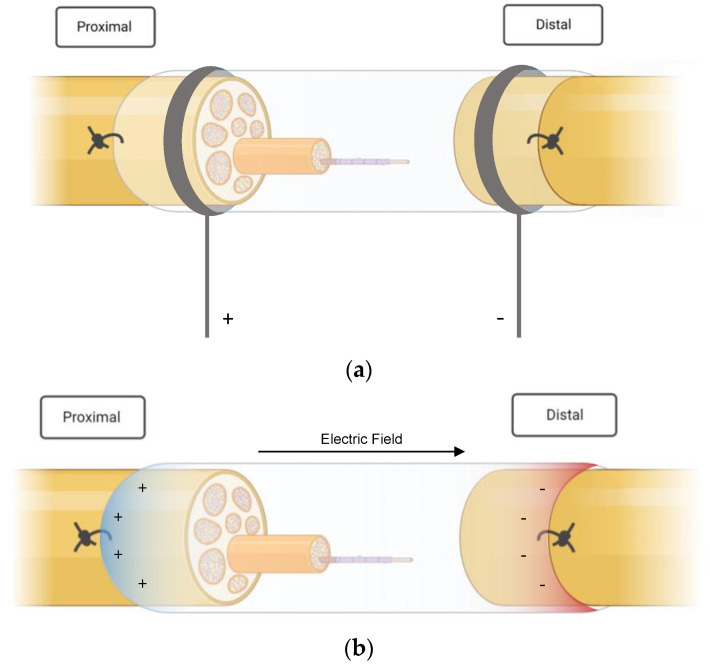
(**a**) External stimulation of the injured nerve. The stumps were sutured into the nerve guidance conduits. (**b**) Self-powered electrical stimulation. The images were created with Biorender.com. https://www.biorender.com (accessed on 17 January 2023).

In four studies, ES was applied directly to the peripheral nerves with needle electrodes in combination with common silicon conduits [24,25,26,27] (see Figure 2a). Liao et al. [25] inserted silicone rubber chambers in the right sciatic nerve of 40 rats that had been treated with taxol (paclitaxel), which is an antineoplastic drug that is known to induce peripheral neuropathy [25]. After the administration and insertion of the NGC, electrical stimulation was performed on 30 rats over three weeks, ten of which did not receive ES treatment and served as a control group. The stimulated animals were further subcategorized into three subgroups according to the frequency of the stimulation: low (2 Hz), medium (20 Hz) and high (200 Hz). A silicone conduit was also used in a study published by Lin et al. [26] in 2014. In this trial, animals received a single shot of streptozotocin prior to surgery to induce diabetes and subsequent peripheral neuropathy. The animals that qualified as diabetic underwent immediate nerve resection and conduit implantation. Stimulation at different currents was performed for three weeks, starting one week after surgery. Three different study groups (C, D and E) were stimulated with 1, 10 and 20 mA, respectively, and compared with a diabetic group (no ES treatment) and a non-diabetic control group after a recovery time of four weeks [26]. The same authors published a similar study with a slightly different approach one year later. Their previous work had investigated the effect of current-modulated ES; the succeeding research focused on the importance of the timing of ES treatment in diabetic peripheral nerve damage. Stimulation at 1 mA was initiated on either day one, day eight or day fifteen [27]. MacEwan et al. [28] examined a way to provide a chronic interface for sciatic nerve stimulation. A polyimide macro-sieve electrode (MSE) was implanted into a silicone conduit. Additionally, glial-derived neurotropic factor (GDNF) was injected in 50% of the MSE-implanted rats to provide potential neurotropic support.

Five studies used polypyrrole (PPy)-based conduits for their experiments [29,30,31,32,33]. Chen et al. [29] fabricated a carboxylic-graphene-oxide-composited polypyrrole/poly-L acid (C-GO/PPy/PLLA) film to create an NGC, which was implanted in a 10 mm nerve gap of Sprague Dawley rats. The sciatic nerve was stimulated through the conduit with a continuous pulse train of 20 Hz and 1 V for one hour per day over one week. A non-stimulated NGC cohort and a group that received an autograft instead of a conduit served as the control [29].

Song et al. [30] opted for less intense electrical stimulation of 100 mV through a polymerizing pyrrole-coated PLCL (L-lactic acid-co-ε-caprolactone) nanofibrous conduit in long-range sciatic defects at the mid-thigh level. Four and eight weeks after applying ES for a total of four times over one week, the rats were examined and compared with the control groups (autograft and non-stimulated NGC groups) [30].

Unlike their colleagues, Sun et al. [31] did not rely on external ES for their research. Instead, they implanted a conductive nerve conduit that provided self-powered electrical stimulation via glucose and oxygen consumption (Figure 2b). The fabrication process entailed polymerization of polypyrrole on the nanofibers of bacterial cellulose in the form of a nerve scaffold, which was then inserted into polycaprolactone (PCL)/chitosan conduits. The prepared conduit provided mechanical stability, adequate flexibility and, most importantly, conductivity. It was reportedly able to form an electrical potential difference of up to 300 mV under the influence of glucose.

As previously mentioned, the lengths of the created nerve gaps varied between the published studies. The longest sciatic nerve defect was reported by H. Zhang et al. [32]. The authors bridged a 20 mm segment with an electrospun lysine-doped PPy/spider silk protein/poly (L-lactic) acid conduit that contained nerve growth factor as an adjuvant to enhance neuronal growth. Early external electrical stimulation was applied with an intensity of 0.6 V and a frequency of 50 Hz starting only 24 h after surgery. This stimulation protocol was carried out for four hours per day for three consecutive days.

In the case of Zhao et al. [33], a polypyrrole/silk fibroin (PPy/SF) conduit was fabricated using 3D bioprinting and electrospinning and was then stimulated with 3 V at 20 Hz.

The amount of time from surgically induced nerve injury to ES treatment varied between the studies. Huang et al. [34] investigated whether delaying electrical stimulation for 2, 4, 12 or 24 weeks impacted the extent of peripheral nerve regeneration in sciatic nerve defects that had been bridged with hollow NGCs. No further details were given concerning the nature or material of the conduit used in this study.

While ES treatment was only applied once in the former trial, Wu et al. [35] administered multiple cycles of stimulation over two weeks. The authors prepared a conductive conduit from hydroxyethyl cellulose (HEC)/soy protein isolate (SPI)/PANI (polyaniline) sponge (HSPS), which was stimulated at 3 V. Furthermore, one of the study groups received brain-derived neurotrophic factor (BDNF), in addition to the HSPS conduit to examine whether the ES could enhance BDNF expression, thereby further promoting nervous tissue regeneration [35]. A similar approach was taken by Li et al. [36] in 2020. They fabricated biodegradable conduits from carbon nanotubes (CNTs) and added sericin, which is a natural protein that was found to have neurotrophic effects [37], to promote functional recovery. One-time electrical stimulation was performed with 3 V at 20 Hz [36].

The use of a deacetyl chitin conduit was reported by one group of authors, namely, Z. Zhang et al. [38]. Electrical stimulation through this biological NGC was applied with 3 V at 20 Hz.

In total, four studies used implantable electric devices in their experiments, making external stimulation unnecessary [28,31,39,40]. Lee et al. [39], who conducted one of the first studies combining NGC and ES, inserted a polymer-based implantable electrode that also served as a conduit and was covered with collagen into rat sciatic nerves at the thigh level to examine the effect of continuous stimulation of 100 Hz (biphasic current) at 20 µA. This implantable device allowed for permanent electrical stimulation for four weeks, after which it was surgically removed. Sun et al. [31] and Wang et al. [40] applied stimulation via a self-electrified device. The conduit in the latter study consisted of galvanic cells made of thin-film magnesium and iron-manganese alloy electrodes on a polymer-based biodegradable material [40].

Details from all included studies are portrayed in Table 2 for a better overview.

The following graphics (Figure 2a,b) illustrate the basic principle of the electrical stimulation performed in the included studies. Essentially, ES protocols can be divided into two groups. Most authors applied ES externally, i.e., via electrodes that were inserted aseptically to avoid contamination and infection [25,26,27,29,30,32,33,34,35,36,38]. Lin et al. [26,27], for example, placed a stainless steel electrode, which was connected to the negative output of a stimulator (cathode (−), into the knee; the anode (+) was positioned in the hip joint. Three authors, on the other hand, created implantable devices that had self-stimulating qualities [31,39,40]. These electroactive stimulators were able to generate an electric field and deliver currents through the nerve stumps without the need for an external stimulation source. A chronically implanted macro-sieve electrode inserted by MacEwan et al. [28] was connected to silicone conduits and stimulated via microwire leads, which were buried subcutaneously.

### 3.2. Outcome Measures

#### 3.2.1. Electrophysiological Tests

The compound muscle action potential (CMAP) and the nerve conduction velocity (NCV) were the quantities most frequently used to evaluate the extent of peripheral nerve regeneration after treatment. Nine out of fifteen authors recorded and compared the NCV values of the study groups and the respective comparative cohorts. The CMAP was measured in six studies, two publications reported the MAP (muscle action potential) and one, namely, Song et al. [30], measured the distal compound muscle potential (DCMAP). MAP, CMAP and DCMAP are all electrophysiological markers for muscle function and represent useful tools in motor nerve conduction studies. The MAP refers to the evoked muscle action potential, whereas the CMAP describes the summation of the action potentials of all stimulated motor endplates measured on one muscle. The DCMAP, which is defined as the distally evoked compound muscle action potential, is often used as a predictor for nerve damage in distal nerve segments [41,42].

In the study by Song et al. [30], the authors reported a significantly higher DCMAP improvement in the ES + PPy and autograft groups compared with the rats that had received conduits without ES treatment after eight weeks. These results coincide with the CMAP findings of Chen et al. [29]. Furthermore, significantly higher NCV scores were achieved in both studies in the electrically stimulated groups [29,30].

According to Liao et al. [25], electrical stimulation through silicone conduits could not achieve a noticeable improvement in taxol-treated rats. However, the MAP area and the amplitude of the regenerated nerves were significantly increased in the low-frequency (2 Hz) group compared with the 20 Hz, 200 Hz and control groups [25]. Significantly higher amplitudes of MAP areas were observed in diabetic rats as well, as observed by Lin et al. [27] in 2015. However, these superior results were only observed in the early onset ES groups (ES first applied on day one or day eight) and could not be confirmed in the delayed ES cohort that was stimulated on day fifteen. The nerve conduction velocity was equally superior in all stimulated rats compared with the non-stimulated animals [27]. In their previous work, the authors found that stimulation of 20 mA (defined as “high” stimulation in this study) yielded better results and led to significantly higher NCV values compared with all other groups (low/medium/no stimulation) except from the non-diabetic rats, which showed significant improvement as well [26].

Contrary to the findings of Lin et al. [27], in a study by Huang et al. [34], delayed ES led to significantly higher CMAP and NCV scores even after a long delay period compared with non-stimulated NGC implanted animals (ES after 2, 4, 12 and 24 weeks). Significantly better electrophysiological results after ES were also reported by Z. Zhang et al. [38] (NCV) and Wu et al. [35] (CMAP). However, the electrically stimulated HSPS conduits in the latter study could not exceed the effect of autografted nerves [35]. Li et al. [36] observed similar results for the CMAP and NCV values. Higher scores were achieved after electrical stimulation of the CNT/sericin group (in the group with a CNT concentration of 0.5) and after autograft implantation [36]. The self-electrified implanted device designed by Wang et al. [40] improved the CMAP in all groups.

#### 3.2.2. Functional Evaluation

A walking track analysis was performed in order to assess motor function recovery and calculate the sciatic function index (SFI). SFI values range from -100 to 0, with a score of 0 indicating healthy motor function and a score of -100 suggesting severe functional impairment [40]. Assessments of SFI scores were reported in seven publications, all of which registered considerable functional improvement after electrical stimulation through nerve conduits [30,31,33,35,36,39,40]. Zhao et al. [33] reported that six months after treatment, rats that had been treated with an electrically stimulated PPy-based conduit showed the best SFI scores out of all the study groups, including the ES group that received a silicon conduit instead of a PPy/SF NGC. Significantly better motor function recovery was recorded in two other trials, which were published by Song et al. [30] and Sun et al. [31], that applied PPy-based material when compared with non-stimulated nerves bridged with NGCs. However, superiority over the effects of autograft implantation could not be shown as the results were similarly significant [30]. The collagen-coated conduit with the implantable electrical stimulator investigated by Lee et al. [39] performed best among all groups in this study, including the ES group without collagen-coated conduits. In carbon nanotube (CNT)/sericin conduits, the group with the higher concentration of CNTs (0.5) and electrical stimulation produced better results than all other cohorts, except for the autograft group [36].

#### 3.2.3. Histopathological and Morphological Findings

After functional and electrophysiological tests were concluded, the dissected nerves were harvested and examined to measure peripheral nerve regeneration. Immunofluorescence and transmission electron microscopy (TEM) was used to assess the changes on a histological basis. The most frequently reported outcome measures for ES + NGC treatment were the myelin sheath thickness, as stated in ten publications, and the axon diameter, as stated in nine publications [29,30,31,32,33,34,35,36,38,40].

Overall, higher myelin sheath thickness was observed in all of the ten studies [29,30,31,32,33,34,35,36,38,40]. Most of the authors reported that the improvement was statistically significant in comparison to the control groups. The only cohort that yielded similar or slightly better results was the autograft group. In accordance with the results of myelin thickness measurements, observation of axon diameter demonstrated significantly more improvement. Chen et al. [29] even registered considerably better results with ES than with autograft implantation.

#### 3.2.4. Muscle Atrophy

A handful of authors investigated the level of muscle reinnervation by harvesting the target muscle (gastrocnemius or triceps muscle) and weighing it [29,30,31,34,36,40]. Denervation of the muscle led to atrophy and lower weight; therefore, an increase in weight or size was interpreted as better reinnervation. Muscle recovery appeared noticeably better in ES groups and autografted animals in all five trials [29,30,31,34,36,40].

A detailed display of the study outcomes is provided in Table 3.

### 3.3. Complications

No serious complications or adverse events were described in the studies; however, some of the authors did not provide any information concerning this issue. Four studies stated that they did not observe any inflammatory response or rejection process [30,32,33,36]. Some authors described the cytotoxicity tests performed on the material, which were ultimately deemed to be unharmful [29,30,31,35,36,40]. A mild, negligible reaction was described in one study [31]. H. Zhang et al. [32] observed a tendency toward self-mutilation in three of the rats with autografts but did not explore this issue further.

## 4. Discussion

The currently available literature concerning peripheral nerve injury treatment via conduit-assisted electrical stimulation was outlined in this systematic review. Even though only fifteen studies were found, a trend toward beneficial effects was observed. Overall, the electrophysiological qualities of damaged nerves seem to improve noticeably under electrical stimulation through nerve conduits, as shown by the highly increased CMAP and NCV scores, which were observed in most of the included studies [26,27,29,30,32,34,35,36,40]. Liao et al. [25] were the only ones who reported no improvement in nerve conduction velocity at all; however, this might be attributed to the fact that the rats had been injected with taxol, which is believed to impair nerve regeneration. It appears that ES enhances functional regeneration as well, as the ES-treated rats showed better functional outcomes in terms of higher SFI scores after treatment [30,31,33,35,36,39,40]. It was noted that in the study by Lee et al. [43], the addition of collagen coating to the stimulated nerve conduit performed better than the ES conduit without collagen, which may be an important aspect in conduit fabrication for future experiments. The extent of muscle recovery was also improved after stimulation, indicating better reinnervation [29,30,34,36,40]. Lastly, TEM revealed beneficial effects of stimulated NGCs for myelin sheath thickness and axon diameter in rat sciatic nerves [29,30,31,32,33,34,35,36,38,40]. Both parameters are signs of axonal recovery, which is the crucial step on the road to nerve regeneration. Overall, the results obtained from this research indicate that treatment with nerve conduits and additional electrical stimulation is beneficial in the regenerative process of peripheral nerve defects in rats and suggested superiority over non-stimulated conduits but not over autografts. However, the NGC + ES outcome was either only slightly inferior to autograft implantation, with no statistical difference or at least comparable. Given the previously outlined drawbacks and complications associated with autografting [7,8], optimizing NGC + ES therapy might provide a suitable alternative to autografts in the future.

### 4.1. ES Protocols

One of the most important observations we made when comparing these studies to one another was the high variation in electrical stimulation protocols with regard to frequency, duration, stimulation onset or conduit type. This was also noticed by ElAbd et al. [44] in 2022. Since ES + NGC treatment is a rather new approach, no standardized guidelines have been established so far. Previously conducted studies suggested that a low-frequency protocol produces better results [45,46], which was also observed by Liao et al. [25], who concluded that a frequency of 2 Hz leads to greater outcome improvements compared with high-frequency stimulation of 200 Hz. The timing might also be an important factor in nerve regeneration. The findings of Lin et al. [27] suggest that ES might be most effective when applied shortly after surgery, which, in their case, was on day one or day eight. A tendency toward early onset for ES was reported by some other authors as well [47]. However, there is also literature that contradicts this theory, such as the study conducted by Huang et al. [34]. In our opinion, the impact of both time and frequency of ES protocols needs to be further evaluated in in vivo studies to provide reliable guidelines. So far, only trends can be observed and no definite recommendations for optimal stimulation protocols can be made.

### 4.2. Limitations

The biggest challenge, in our opinion, is the variety of treatment protocols. For one, many different materials were used to fabricate nerve conduits. Furthermore, proteins, growth factors or other supporting substances were added during the manufacturing process, further decreasing the comparability of the study results. As previously mentioned, high variation concerning frequency, duration and onset of stimulation was observed as well. In addition, some of the authors used self-electrified implants without giving explicit details concerning ES parameters delivered by said implants [28,40]. Another aspect worth mentioning is the heterogeneity concerning the sciatic nerve transection site, as portrayed in Table 2. While only a small number of authors provided detailed information on this subject, a high variety was observed among the few studies that reported on the location of the created nerve defect. We recommend reporting and standardizing the transection level for future experiments. Lastly, authors sometimes used different parameters to evaluate the study outcome, which poses a challenge in comparing the efficacy of the applied therapy reported in different studies. We focused on the outcome measures that had been investigated in the majority of the studies, which were mostly motoric qualities. More research focusing on the effect of NGC + ES on sensory parameters in addition to motor function would provide a more comprehensive understanding of the efficacy of this therapeutic approach in nerve regeneration.

To conclude, standardized study conditions and treatment protocols should be appointed for future research on this topic to increase comparability among different trials and hopefully prove the efficacy of the procedure.

## 5. Conclusions

In conclusion, the studies presented in this work delivered promising results for electrical stimulation through nerve conduits in peripheral nerve defects in in vivo experiments. NGC + ES therapy showed great potential in improving nerve regeneration and nerve function after peripheral nerve injury in rat models. Although a positive trend toward treatment efficacy can be observed in the current literature, more research is warranted to confirm this hypothesis.

A high variety of ES protocols and conduit material was observed. Standardized protocols are necessary to improve the outcome reliability and hopefully establish guidelines for future clinical use. 

## Figures and Tables

**Table 1:** Search strategy.

Search Terms	OVID (Embase, Medline, Cochrane Library)	PubMed
Nerve conduit (1)	[1 AND 3]	[1 AND 3]
Nerve guidance conduit (2)	OR [1 AND 4]	OR [1 AND 4]
Electrical stimulation (3)	OR [1 AND 5]	OR [2 AND 3]
Electrically conducting (4)	OR [2 AND 3]	OR [2 AND 4]
Electrically conductive (5)	OR [2 AND 4]	
	OR [2 AND 5]	

**Table 2:** Study Details.

Authors, Year.	N	Nerve Defect	NGC Type	ES Protocol	Follow-Up	(Comparative/Control) Groups	
Chen et al. [29], 2019	27	Sciatic nerve, 10 mm	C-GO/PPy/PLLA	1 V, 20 Hz 1 h/day, 7 days 30 min/day for 3 weeks	4, 8, 12 weeks post-treatment	Autograft, same NGC-ES	
Huang et al. [34], 2013	192	Sciatic nerve, 5 mm	Hollow NGC	Delayed ES: after 2, 4, 12, 24 weeks at 3 V, 20 Hz, 1 × 20 min	4, 12, 24 weeks	NGC-ES	
Lee et al. [39], 2010	27	Sciatic nerve, 7 mm; thigh level	Polyimide-based collagen -coated conduit, implanted electrical stimulator	20 µA, 100 Hz, 100 µs duration, for 4 weeks continuously	2, 4 weeks	NGC no collagen (I), NGC collagen (II), NGC no collagen + ES (III), NGC collagen + ES (IV)	
Li et al. [36], 2020	85–90	Sciatic nerve, 10 mm	CNT/sericin NGC	3 V, 20 Hz, 0.1 ms, 1 × 1 hour	8, 12 weeks	0 mg/mL concentration, 0.5 mg/mL, 0 mg/mL + ES, 0.5 mg/mL − ES, AG	
Liao et al. [25], 2020	40	Sciatic nerve, 10 mm *	Silicone rubber NGC	1 mA, 2/20/200 Hz, 3 times/week for 3 weeks	4 weeks	Low vs. medium vs. high ES vs. no ES	
Lin et al. [26], 2014 (diabetic rats)	50	Sciatic nerve, 10 mm	Silicone rubber NGC	1/10/20 mA, 2 Hz, 100 µs, 15 min, every other day for 3 weeks	4 weeks	1 mA vs. 10 mA vs. 20 mA ES (# groups C–E) vs. no ES (B#) vs. non-diabetic NGC without ES (A)	
Lin et al. [27], 2015 (diabetic rats)	50	Sciatic nerve, 10 mm	Silicone rubber NGC	1 mA, 2 Hz, 100 µs, 15 min every other day for 2 weeks	-	ES at day 1 (A#) vs. day 8 (B#) vs. day 15 (C#) vs. no ES# (D) vs. non- diabetic no ES (E)	
MacEwan et al. [28], 2016	40	Sciatic nerve, 4 mm	Silicone NGC + MSE	-	-	I: control, II: saline, III: GDNF, IV: MSE + saline, V: MSE + GDNF	
Song et al. [30], 2016	30	Sciatic nerve, 15 mm; mid-thigh	PPY/PLCL	0.1 V, 1 hour/day, 4 times, for 1 week period	3 months	Autograft, same NGC-ES	
Sun et al. [31], 2019	20	Sciatic nerve, 15 mm; lower limb	Pt-BC/PPY-N-CNT (self -powered ES)	Up to 0.3 V	4, 8 weeks	Non-stimulating NGC	
Wang et al. [40], 2020	55	Sciatic nerve, 10 mm; hind limb	PCL PLLA-PTMC bilayer conduit with galvanic cells (self-electrified)	-	4, 8, 12 weeks	Mg group, FeMn group, hollow NGC, autograft	
Wu et al. [35], 2020	50	Sciatic nerve, 10 mm; lateral thigh	HSPS conduit	3 V, 1 hour every other day, 7 times total	3, 9, 12 weeks	HSPS, HSPS with electrodes but no ES, HSPS + BDNF, AG	
Zhang H. et al. [32], 2015	24	Sciatic nerve, 20 mm	L-PRPN	0.6 V, 50 Hz, 2 × 2 h/day, for 3 days	3 months	Same NGC-ES, natural regeneration group	
Zhang Z. et al. [38], 2014	32	Sciatic nerve, 2 mm **	Deacetyl chitin conduit	3 V, 20 Hz, 0.1 ms 1 × 1 hour	10 months	Same NGC-ES	
Zhao et al. [33], 2020		Sciatic nerve, 10 mm; lateral thigh	PPY/SF	3 V, 20 Hz, 1 hour, every other day, 7 times total	6, 12 weeks	PPy-ES, PPY + ES, silicone-ES, silicone + ES, AG	

N: total number of animals; NGC + ES: nerve guidance conduit with electrical stimulation; NGC-ES: nerve guidance conduit without electrical stimulation; AG: autograft; PPY: polypyrrole; C-GO/PPy/PLLA: carboxylic-graphene-oxide-composited polypyrrole/poly-L-lactic acid; CNT/sericin: carbon nanotube/sericin conduit; MSE: macro-sieve electrode; GDNF: glial-derived neurotropic factor; PPY/PLCL: polymerizing pyrrole-coated poly(l-lactic acid-co-ε-caprolactone); Pt-BC/PPY-N-CNT: platinum (nanoparticles)-bacterial cellulose/PPY-nitrogen-doped CNT; PCL PLLA-PTMC: polycaprolactone copolymer of poly(l-lactic acid) and poly(trimethylene carbonate); Mg: magnesium; FeMn: iron-manganese; HSPS: hydroxyethyl cellulose (HEC)/soy protein isolate (SPI)/PANI(polyaniline) sponge; BDNF: brain-derived neurotrophic factor; PPY/SF: polypyrrole/silk fibroin; * nerve defect between knee and hip joint; ** nerve defect 1 cm proximal to the sciatic bifurcation.

**Table 3:** Outcomes.

Authors, Year	CMAP	NCV	SFI	Muscle Recovery (Weight)	Myelin Sheath Thickness	Axon Diameter
Chen et al. [29], 2019	Higher in m NGC + ES and autograft at 8 weeks *	Higher than NGC-ES at 12 weeks *	-	Higher than NGC-ES at 12 weeks * (musculus gastrocnemius)	Higher than NGC-ES *	Higher than NCG-ES * and AG groups
Huang et al. [34], 2013	Higher in ES groups *	Higher in ES groups *	-	Higher in ES groups * (m. gastrocnemius)	Higher in ES groups *	Higher in ES groups *
Lee et al. [39], 2010	-	-	Higher in group IV at 4 weeks *	-	-	-
Li et al. [36], 2020	Highest in 0.5 + ES, AG, 0.5 − ES at 12 weeks	Highest in 0.5 + ES and AG (at 8 weeks*)	Higher in 0.5 + ES vs. 0−ES * vs 0.5−ES, 0 + ES	Higher in 0.5 + ES vs. all * but AG	Twice as high in 0.5 + ES group vs. all but AG	Higher with 0.5 + ES * and AG * groups at 12 weeks
Liao et al. [25], 2020	MAP: peak amplitude highest with low ES *	No improvement using ES	-	-	-	-
Lin et al. [26], 2014 #	-	Higher in groups D * and A *	-	-	-	-
Lin et al. [27], 2015 #	MAP area: A and B higher than D *	Higher in groups A–C * and E *	-	-	-	-
MacEwan et al. [28], 2016	-	-	Improved SFI	-	-	-
Song et al. [30], 2016	Higher in NGC + ES and AG at 8 weeks * (DCMAP)	Higher than NGC−ES *; comparable to AG at 4, 8 weeks	Higher in NGC + ES and AG at 4 weeks *	Higher in NGC + ES and AG * (m. triceps)	Higher in NGC + ES and AG at 4 weeks *	Higher in NGC + ES and AG * groups
Sun et al. [31], 2019	-	-	Higher in stimulated group *	-	Higher in stimulated group *	Higher in stimulated group *
Wang et al. [40], 2020	Improved in all groups	-	Higher with ES than other groups after 4 weeks * except AG	Higher in ES and AG * (m. gastrocnemius)	Higher with ES than in FeMn and hollow * group	Higher with ES than all groups * except AG
Wu et al. [35], 2020	Higher in ES than all other groups * except AG	-	Higher in ES than all other groups * except AG (similar)	-	Highest in ES * and AG * group	Highest in ES * and AG * groups
Zhang H. et al. [32], 2015	Best results with ES	Best results with ES	-	-	Best results with ES	-
Zhang Z. et al. [38], 2014	-	Higher with ES at 6 *, 12 weeks	-	-	Higher with ES at 6, 12 weeks	Higher with ES at 6, 12 weeks *
Zhao et al. [33], 2020	-	-	Best results in PPY + ES group after 6 months	-	Best results in PPY + ES and AG group	Best results in PPY + ES and AG groups

* statistically significant results #: diabetic rats; (D)CMAP: (distal) compound muscle action potential; NCV: nerve conduction velocity; SFI: sciatic function index; NGC + ES: nerve guidance conduit with electrical stimulation; NGC-ES: nerve guidance conduit without electrical stimulation; AG: autograft; FeMn: iron-manganese; PPY: polypyrrole;

## Data Availability

The data that support the findings of this study was obtained from online databases (PubMed, Ovid), journal websites, or other research platforms where restrictions or charges may apply. Such a dataset may be requested from the respective journals or by contacting the authors directly.
